# Finding the Optimal Imputation Strategy for Small Cattle Populations

**DOI:** 10.3389/fgene.2019.00052

**Published:** 2019-02-18

**Authors:** Paula Korkuć, Danny Arends, Gudrun A. Brockmann

**Affiliations:** Animal Breeding Biology and Molecular Genetics, Albrecht Daniel Thaer-Institute for Agricultural and Horticultural Sciences, Humboldt University of Berlin, Berlin, Germany

**Keywords:** cattle, DSN, Holstein, sequencing, 1000 Bull Genomes Project, SNP, SNP chip, imputation

## Abstract

The imputation from lower density SNP chip genotypes to whole-genome sequence level is an established approach to generate high density genotypes for many individuals. Imputation accuracy is dependent on many factors and for small cattle populations such as the endangered German Black Pied cattle (DSN), determining the optimal imputation strategy is especially challenging since only a low number of high density genotypes is available. In this paper, the accuracy of imputation was explored with regard to (1) phasing of the target population and the reference panel for imputation, (2) comparison of a 1-step imputation approach, where 50 k genotypes are directly imputed to sequence level, to a 2-step imputation approach that used an intermediate step imputing first to 700 k and subsequently to sequence level, (3) the software tools Beagle and Minimac, and (4) the size and composition of the reference panel for imputation. Analyses were performed for 30 DSN and 30 Holstein Frisian cattle available from the 1000 Bull Genomes Project. Imputation accuracy was assessed using a leave-one-out cross validation procedure. We observed that phasing of the target populations and the reference panels affects the imputation accuracy significantly. Minimac reached higher accuracy when imputing using small reference panels, while Beagle performed better with larger reference panels. In contrast to previous research, we found that when a low number of animals is available at the intermediate imputation step, the 1-step imputation approach yielded higher imputation accuracy compared to a 2-step imputation. Overall, the size of the reference panel for imputation is the most important factor leading to higher imputation accuracy, although using a larger reference panel consisting of a related but different breed (Holstein Frisian) significantly reduced imputation accuracy. Our findings provide specific recommendations for populations with a limited number of high density genotyped or sequenced animals available such as DSN. The overall recommendation when imputing a small population are to (1) use a large reference panel of the same breed, (2) use a large reference panel consisting of diverse breeds, or (3) when a large reference panel is not available, we recommend using a smaller same breed reference panel without including a different related breed.

## Introduction

Imputation from lower density SNP chip genotypes to whole-genome sequencing level is a practical, cheap and fast method to generate high density genotypes for many individuals. While imputing, missing values of lower density genotypes of a target population are inferred using a reference panel consisting of genotypes from higher resolution data. The variety of existing methods for imputation is large (Marchini and Howie, 2010), from the many software tools available for imputation we used Beagle (Browning and Browning, 2016) and Minimac (Das et al., 2016) which exploit haplotype patterns for imputation.

In cattle breeding, most animals from breeds with limited population sizes currently are genotyped with low density (3 k or 10 k) or medium density (50 k) SNP chips, but rarely with high density (700 k) SNP chips and far less animals are whole-genome sequenced. Therefore, most imputation studies have been performed in breeds such as Holstein Friesian (HF), where a relatively high number of high density genotyped or whole-genome sequenced animals are available. For example, the 1000 Bull Genomes Project^1^ offers a large reference panel including many HF animals on sequence level that can be used for imputation within the same breed (Brøndum et al., 2014; van Binsbergen et al., 2015; Pausch et al., 2017). However, imputation to higher density is especially challenging in populations where only a low number of high density genotypes or sequence data is available. One such population is the endangered German Black Pied cattle (DSN, “Deutsches Schwarzbuntes Niederungsrind”) which is considered to be one of the founder breeds of German HF (Porter, 1991). The population of DSN consists of about 2000 pure-bred animals and only 30 of them were whole-genome sequenced, while many 50 k genotypes are available for this breed. Thus, finding an optimal imputation strategy for such small populations is necessary in order to obtain high quality imputed genotypes for further analyses.

Most importantly, the accuracy of imputation has to be sufficiently high to allow for reliable conclusions in further analyses, such as genome-wide association studies and identification of causal DNA variants (Li et al., 2009; Marchini and Howie, 2010). Several factors are known to influence the accuracy of imputation: It was shown that the imputation accuracy improved by increasing the number of animals in the reference panel for imputation and also if the reference panel was composed of close relatives of the target population (Sargolzaei et al., 2014; van Binsbergen et al., 2014; Pausch et al., 2017; Wang and Chatterjee, 2017). Furthermore, a 2-step imputation strategy that first imputes from an initial lower density to an intermediate higher density and afterwards to the desired density was reported to provide more accurate imputation compared to direct imputation to the desired density (VanRaden et al., 2013; van Binsbergen et al., 2014; Kreiner-Møller et al., 2015). Nevertheless, for breeds consisting of a limited number of individuals there is no recommended strategy for reaching optimal imputation accuracy.

In this paper, we investigated multiple imputation strategies with regard to phasing of the target population and reference panel, size and composition of the reference panel for DSN animals as an example for small populations. We compared the imputation tools Beagle and Minimac and a 1-step imputation to the previously recommended 2-step imputation approach. We used data from the 1000 Bull Genomes Project which includes 30 sequenced DSN animals and compared the analyses to a set of 30 randomly chosen sequenced HF animals from the 1000 Bull Genomes Project in order to exclude breed-specific effects. Results for HF are provided in the Supplementary Information.

## Materials and Methods

### Genomic Data for Imputation

Whole-genome sequencing data of 2,333 *Bos taurus* animals was provided as raw SNP calls and as Beagle imputed phased SNP calls by the 1000 Bull Genomes Project (Run 6.0) (see Footnote 1) (Daetwyler et al., 2014). Additional filtering of the dataset was performed by removing animals which (1) had no breed information, (2) were crossbreeds, or (3) belonged to breeds with less than 10 animals. Genetic similarity was calculated using pairwise relative Manhattan distances (Equation 1). Animals with high genetic similarity (>0.99) not explained by kinship were removed from the dataset. The resulting dataset contained 2,145 animals from 30 breeds, including 541 HF and 30 DSN animals. In the following, we denote the set of these 2,145 animals as “1000 bulls.”

SNPs that were not polymorphic in DSN or HF were removed from the dataset leading to a total of 22,179,359 SNPs. SNP chip probe sequences of both the Illumina^®^ Bovine50SNP chip (50 k SNP chip) and the Illumina^®^ BovineHD Genotyping BeadChip (700 k SNP chip) were remapped against the *Bos taurus* genome version UMD3.1 (Zimin et al., 2009) using NCBI blastn (Altschul et al., 1990) in order to obtain genome positions for SNPs on the same genome version as the “1000 bulls” dataset. SNP probes that mapped to multiple genomic locations (1,617 from the 50 k SNP chip and 7,086 from the 700 k SNP chip) were excluded from further analyses.

Lower density datasets serving as target populations or reference panels for subsequent imputation were generated *in silico* by downscaling the “1000 bulls” datasets to the level of the 50 k and/or 700 k SNP chip. Only SNPs were used that occurred in sequencing data and in the remapped 50 k or 700 k SNP chip data, respectively. After the harmonization between SNP chips and sequencing data, the 50 k downscaled datasets consisted of 47,272 SNPs and the 700 k downscaled data of 649,124 SNPs.

### Target Populations and Reference Panels for Imputation

The 30 sequenced DSN animals from the “1000 bulls” dataset were scaled down to 50 k SNP chip level and used as target population for imputation. The “1000 bulls” dataset was used as reference panel for imputation. We investigated if the number of animals in the reference panel and the relatedness of the reference panel with the target population affects the imputation accuracy. Therefore, not only the “1000 bulls” reference panel, but also a reference panel consisting of 30 DSN was used for imputation.

### Imputation Software and Accuracy Estimation

The software tools Beagle (version 4.1, 21/01/2017) (Browning and Browning, 2016) and Minimac3 (version 2.0.1, 06/2016) (Das et al., 2016) were used for imputation. Beagle, a parallelized and memory efficient software tool that uses linear interpolation to impute ungenotyped variants, was used with its default settings. For Minimac, a computationally efficient software implementation of the Markov Chain Haplotyping (MaCH) algorithm (Li et al., 2010) for genotype imputation, the GT format parameter for estimating the most likely genotype was used.

Imputation accuracy was evaluated by leave-one-out cross validation using the 30 sequenced DSN animals as target population. Leave-one-out means that each to be imputed animal was removed from the reference panel and the remaining animals were retained in the reference panel for imputation. This was repeated for each of the 30 DSN animals.

Imputation accuracy between observed and imputed genotypes was accessed for the number of SNP variants *i = 1*, …, *n* on sequence level using three measurements to allow for comparisons to other studies: (1) Percentage of correctly imputed genotypes (percent identity), (2) Pearson’s correlation coefficient *r* (Druet et al., 2010; Brøndum et al., 2014; van Binsbergen et al., 2014; Pausch et al., 2017), and (3) relative Manhattan distance *d_M_* (Zhang and Druet, 2010), which corresponds to the percentage of correctly imputed alleles. Relative Manhattan distance was calculated as described in Equation 1:

$$d_{M}=1-\frac{\Sigma_{i=1}^{n}|{\text{observed genotypes}}_{i}-{\text{imputed genotypes}}_{i}|}{2n}$$

Genotypes were coded as 0, 1, and 2 corresponding to homozygous reference allele, heterozygous, and homozygous alternative allele, respectively, whereas the reference allele was determined based on the UMD3.1 reference genome.

The results and corresponding figures in this study are presented using the relative Manhattan distance, and measurements calculated using the percent identity and correlation are provided in the Supplementary Tables 2–5. Please, note that the results and conclusions are robust with respect to the imputation accuracy measurements used.

To reduce runtime and computational resources, leave-one-out cross validation was performed for chromosomes 1 to 5 out of the 29 autosomes of the *Bos taurus* genome. The results are consistent with results obtained when all autosomes were used (data not shown).

### Phasing Strategies of Target and Reference Datasets

The imputation accuracy was tested with regard to different phasing strategies of the target population and reference panel. Reference panels were phased with Beagle 4.1 (Browning and Browning, 2007) or Eagle 2.3.4 (Loh et al., 2016). The Beagle phased reference panel was provided by the 1000 Bull Genomes Project. The Eagle phased reference panel was generated from the raw SNP calls of the 1000 Bull Genomes Project. For consistency, the raw dataset was filtered to include only SNPs that are also in the Beagle phased reference panel, and SNPs were subsequently phased using Eagle. The target populations consisting of 30 DSN cattle were generated from the reference panels by downscaling them to 50 k level. Three different target populations on 50 k level were used for imputation: An unphased target population, a target population phased with Beagle, and a target population phased with Eagle.

The effect of phasing on the imputation accuracy was evaluated using chromosome 1. Since the highest imputation accuracy was found when the target population and reference panel were phased with Beagle, Beagle phased data was used for subsequent analyses.

Statistical significance of the imputation accuracy with respect to phasing of the target population and of the reference panel was assessed using the following ANOVA model:

$$\text{Imputation accuracy=}{Pha\,\sin g}_{\text{target population}}+{Pha\,\sin g}_{\text{Reference panel}}$$

ANOVA was performed separately for each target population (DSN, HF), imputation software (Beagle, Minimac), and composition of the reference panel (30 DSN or “1000 bulls”). *p*-values < 0.05 were considered significant.

### Imputation Approaches

**FIGURE 1:**
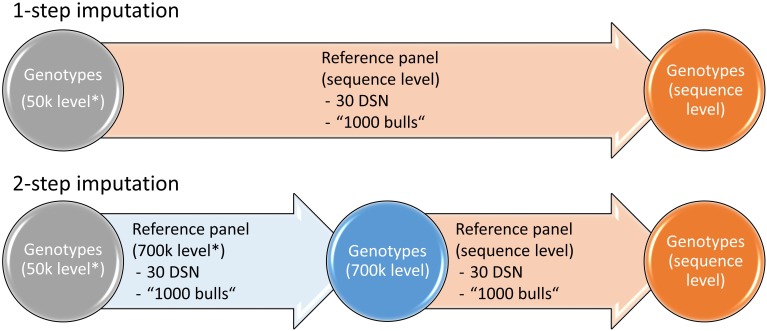
Imputation strategies for the 1- and 2-step imputation approaches. While in the 1-step imputation the genotypes are directly imputed from 50 k to sequence level, in the 2-step imputation the genotypes are first imputed from 50 to 700 k level after which imputation is performed to sequence level. Either the 30 DSN or the “1000 bulls” reference panel were used for imputation. Genotypes that were scaled down to 50 k or 700 k level are labeled with “^∗^”. Similar colors refer to genotypes of the same 50 k (gray), 700 k (blue) or sequence (orange) level.

To investigate the effect of the imputation approach on the imputation accuracy, we compared the 1-step versus the 2-step imputation approach (Figure 1). In the 1-step approach, the target population that was downscaled to 50 k level was imputed to sequence level. In the 2-step approach, the target population was first imputed from 50 to 700 k level using a reference panel that was scaled down to 700 k level and subsequently from 700 k to sequence level using a reference panel on sequence level.

Statistical significance of imputation accuracy between software tools (Beagle and Minimac), imputation approaches (1-step imputation, 2-step imputation), and reference panels (30 DSN, 1000 bulls) was assessed using pairwise *t*-tests for each target population (DSN, HF) separately. *p*-values < 0.05 after multiple testing correction using Bonferroni were considered significant.

### Imputation Performance for HF Animals

To exclude any breed-specific effects on the results, the phasing and imputation approaches were tested also for a set of 30 randomly chosen HF animals from the “1000 bulls” dataset. The 30 HF cattle were used either on 700 k or sequence level as a reference panel or downscaled to 50 k as a target population. Every procedure performed for the 30 DSN target population was also performed for the target population consisting of 30 HF animals. Results using the 30 HF target population were consistent with the DSN population and are provided in the Supplementary Information.

## Results

### Phasing of the Target Population and Reference Panel Affects Imputation Accuracy

**FIGURE 2:**
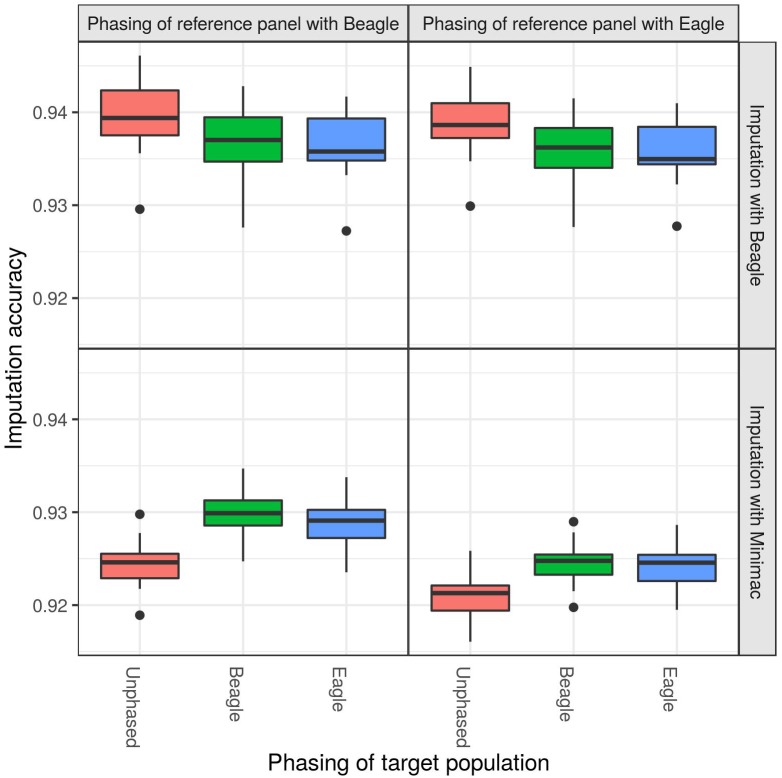
Comparison of different phasing strategies. The target population consisting of 30 DSN animals was either unphased (red), phased with Beagle (blue) or phased with Eagle (green), while the “1000 bulls” reference panel was phased with Beagle (left panel) or Eagle (right panel). Imputation was performed using Beagle (top panel) or Minimac (bottom panel) from 50 k to sequence level and imputation accuracy was calculated as relative Manhattan distance. Imputation with Beagle showed the best accuracy when using an unphased target population. The highest accuracy for imputations with Minimac was observed when both the target population and reference panel were phased with Beagle.

When imputing the DSN target population with the “1000 bulls” reference panel from 50 k to sequence level, significant differences in imputation accuracy were observed with regard to different phasing strategies (Figure 2 and Supplementary Table 1). While imputing with Beagle, the best mean imputation accuracy was reached when the target population was unphased (ANOVA *p*-value = 1.0E-07), regardless whether the reference panel was phased using Beagle (94.0%) or Eagle (93.9%). Imputation accuracy decreased by around 0.3% when the target population was phased with Beagle or Eagle compared to being unphased (*t*-test *p*-value = 5.7E-08). Also the imputation with Minimac showed significant differences with regard to the phasing of the target population (ANOVA *p*-value = 1.5E-24) and reference panel (ANOVA *p*-value = 3.1E-31) (Supplementary Table 1). The highest mean imputation accuracy (93.0%) was observed when the target population and the reference panel were both phased with Beagle (Figure 2 and Supplementary Table 2). In contrast to the imputation with Beagle, Minimac showed the lowest mean imputation accuracy when an unphased target population was used (92.1%).

Using the 30 DSN reference panel, imputation with Beagle showed no difference in accuracy for different phasing strategies of the target population and reference panel (Supplementary Tables 1, 2 and Supplementary Figure 1). However, using the 30 DSN reference panel did not change the Minimac results, albeit the imputation accuracy was lower (mean accuracy 93.0% for “1000 bulls” reference panel compared to 91.5% for 30 DSN reference panel).

The imputation results for the target population consisting of 30 HF animals are consistent with the findings using the 30 DSN as target population (Supplementary Tables 1, 2 and Supplementary Figures 2, 3). Therefore, we are confident that the results obtained are independent of the breed under investigation.

Since the imputation accuracy for target populations and reference panels that were both phased with Beagle showed the overall best performance, independent of the imputation software used, we decided to use data phased using Beagle for further analyses.

### Beagle Achieves Higher Imputation Accuracy Using a Large Reference Panel

**FIGURE 3:**
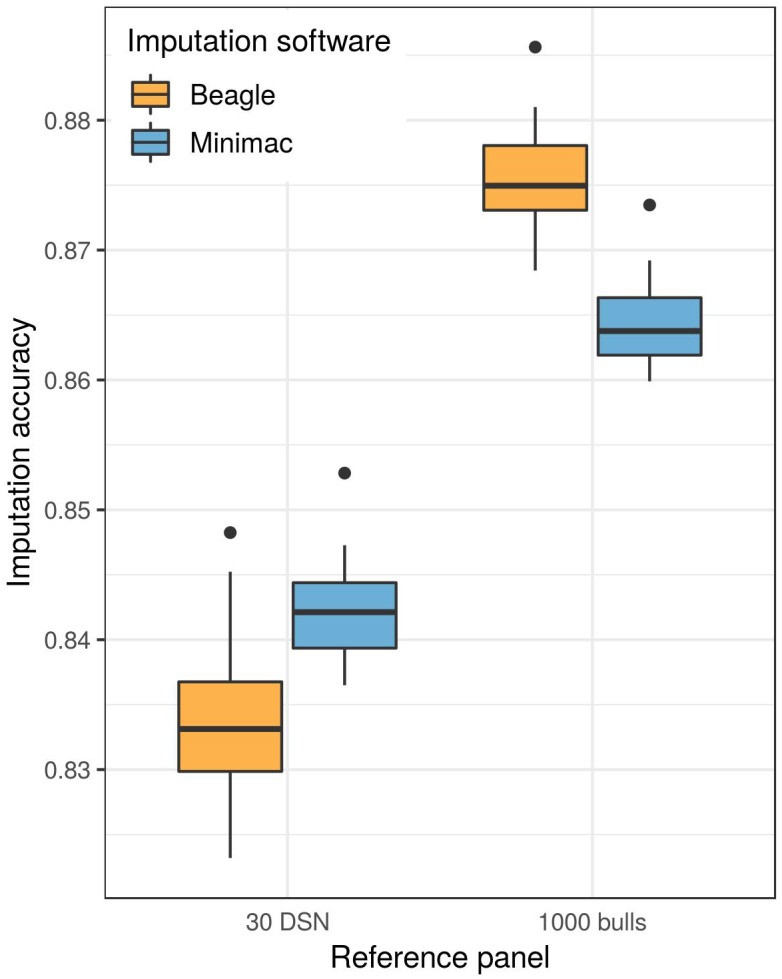
Imputation software comparison for the imputation of the DSN target population. Either the 30 DSN or the “1000 bulls” reference panel was used for imputation from 50 k to sequence level and imputation accuracy was calculated as relative Manhattan distance. We observed a significantly higher imputation accuracy with Beagle (orange) than with Minimac (blue) using the “1000 bulls” reference panel (*t*-test *p*-value = 8.0E-27). The accuracy of imputation with the smaller reference panel (30 DSN) was significantly higher when imputing with Minimac (*t*-test *p*-value = 1.3E-04).

A higher mean imputation accuracy was reached with Minimac (91.0%) compared to Beagle (90.6%, *t*-test *p*-value = 1.3E-04) when the 30 DSN reference panel was used for the imputation of the DSN target population from 50 k to sequence level (Figure 3 and Supplementary Table 3). In contrast, when imputing using the “1000 bulls” reference panel, Beagle (93.2%) showed significantly better performance compared to Minimac (92.4%, *t*-test *p*-value = 8.0E-27).

The same results were observed for the target population consisting of 30 HF using the “1000 bulls” reference panel for imputation (*t*-test *p*-value = 8.6E-22) (Supplementary Table 3 and Supplementary Figure 4). Different from the DSN target population, no difference was found between Beagle and Minimac when using the 30 HF reference panel for the HF target population.

Overall, the accuracy of imputation is higher when using a bigger reference panel even if it consists of diverse breeds which is the case in the “1000 bulls” reference panel (92.8% for DSN target population, 94.1% for HF target population) compared to using a small reference panel of animals from the same breed (90.8% for DSN target population, 91.1% for HF target population) regardless of the imputation software (Supplementary Table 3).

Since Beagle imputed genotypes overall with a higher accuracy in comparison to Minimac, further analyses were done with Beagle. In addition, imputation of a single animal from 50 k to sequence level with the “1000 bulls” reference panel using Beagle was around 24 times faster compared to the imputation using Minimac.

### 1-Step Imputation Performs Better Than 2-Step Imputation

**FIGURE 4:**
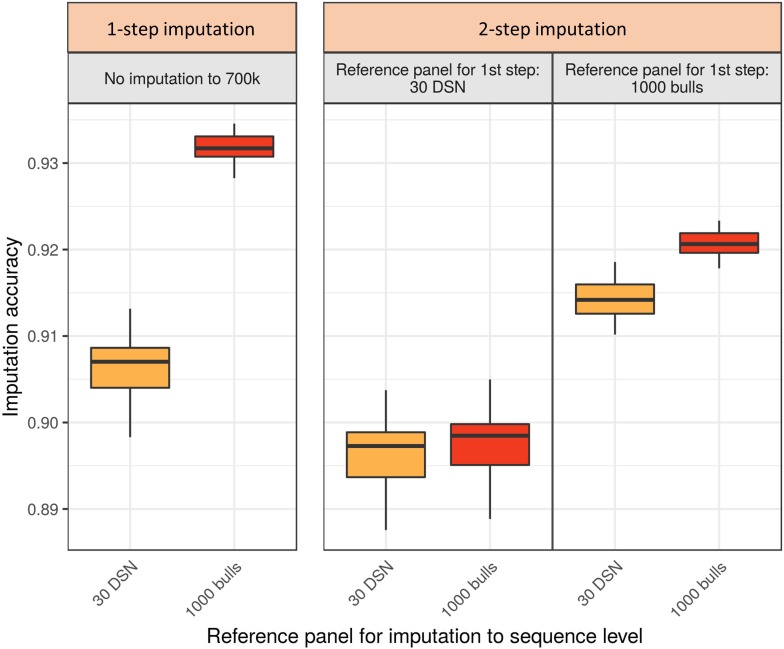
Comparison of imputation accuracy between the 1-step and 2-step imputation approach using Beagle for the DSN target population. Two reference panels (30 DSN and “1000 bulls”) were used during the 1-step imputation from 50 k to sequence level. The same two panels were used for the first (50 to 700 k) and the second step (from 700 k to sequence level) in the 2-step imputation approach. The imputation accuracy was calculated as relative Manhattan distance. 1-step imputation using the “1000 bulls” reference panel provided the best mean imputation accuracy (93.2%). The colors refer to the reference panels used for the imputation to sequence level (yellow – 30 DSN reference panel, orange – “1000 bulls” reference panel).

Imputing the DSN target population with the “1000 bulls” reference panel, the highest mean accuracy of 93.2% was observed for the 1-step imputation approach (Figure 4 and Supplementary Table 4). For comparison, the highest mean accuracy using the 2-step imputation approach was significantly lower (92.1%, *t*-test *p*-value = 3.9E-34). In the 2-step imputation approach the “1000 bulls” reference panel was used twice, once scaled down to 700 k level used for the imputation from 50 to 700 k level (first step) and second on sequence level for the subsequent imputation from 700 k to sequence level (second step).

If differently sized and composed reference panels were available for the first and second step of the 2-step imputation approach, the imputation accuracy was significantly affected (Figure 4 and Supplementary Table 4). If only a small reference panel (30 DSN) was available for the first step of the 2-step imputation approach, then using a bigger reference panel (“1000 bulls”) for the second step did not increase the overall imputation accuracy. Hence, the mean imputation accuracies were 89.6% or 89.7% when the 30 DSN or “1000 bulls” reference panel were used in the second step, respectively. Interestingly, when many animals were available for the imputation to 700 k level (“1000 bulls” reference panel) and only few animals for the final imputation to sequence level (30 DSN reference panel), higher mean accuracy was obtained in the 2-step (91.4%) compared to the 1-step imputation approach using the 30 DSN reference panel (90.6%; *t*-test *p*-value = 9.0E-13). The same qualitative results were observed when comparing the 1-step versus 2-step imputation approach for the HF target population (Supplementary Table 4 and Supplementary Figure 5).

### Using a Wrong Reference Panel Can Hurt Imputation Accuracy

**FIGURE 5:**
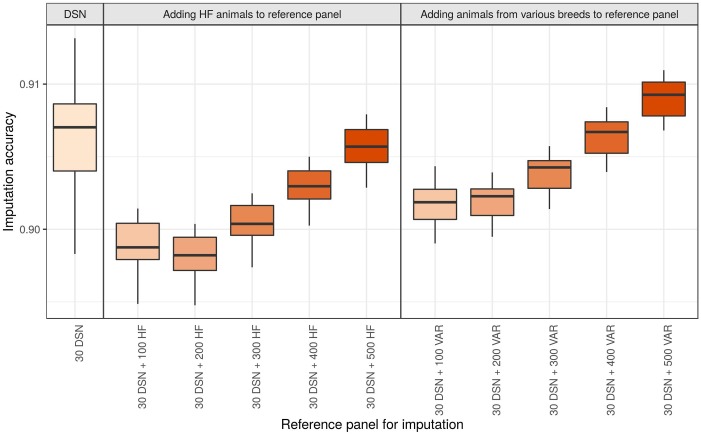
Comparison of imputation accuracy with regard to differently sized and composed reference panels for the DSN target population. The imputation was performed from 50 k to sequence level with Beagle using the 30 DSN reference panel and successively adding 100 HF animals or animals from various breeds (VAR) to the initial 30 DSN reference panel. The imputation accuracy was calculated as relative Manhattan distance. We observed a decrease in imputation accuracy for DSN when animals distant from DSN are added to the reference panel even when the size of the reference panel increased. Interestingly, the drop in imputation accuracy was bigger when adding HF animals than when adding animals from various breeds. The intensity of the color refers to the total number of animals in the reference panel.

Genotype imputation from 50 k to sequence level of the DSN target population using the 30 DSN reference panel achieved a mean imputation accuracy of 90.6%. Surprisingly, when we added 100 additional HF animals (randomly chosen from the “1000 bulls” reference panel) to the reference panel, we observed a significant reduction in mean imputation accuracy to 89.9% (*p*-value = 1.4E-11) (Figure 5 and Supplementary Table 5). When continuing to add 100 HF animals (up to 500 HF animals) to the reference panel, we observed that the imputation accuracy increased with increasing number of HF animals in the reference panel. Nevertheless, the mean imputation accuracy remained significantly lower compared to the original imputation accuracy using the 30 DSN reference panel unless 500 HF were added to the reference panel (200 HF: 89.8%, 300 HF 90.0%, 400 HF: 90.3%, 500 HF: 90.6%). To investigate if the decrease in accuracy is also observable when animals from various breeds were used for imputation, we performed the same analysis now adding 100 randomly chosen animals from various breeds out of the “1000 bulls” reference panel (Figure 5 and Supplementary Table 5). Again, we observed a significant drop in mean imputation accuracy when 100 additional animals from various breeds were added, compared to using the 30 DSN reference panel (*p*-value = 4.8E-06). However, the drop in accuracy was significantly higher when adding 100 HF animals (–0.7%) than 100 animals from various breeds (–0.4%) to the reference panel (*p*-value = 2.7E-08). Imputation accuracy was restored to the level of the 30 DSN reference panel if the reference panel contained 30 DSN and 300 animals from various breeds. We observed the same results for the target population consisting of 30 HF when successively adding 100 animals from various breeds to the reference panel (Supplementary Table 5 and Supplementary Figure 6).

## Discussion

Due to their limited population size, DSN animals are not unrelated and family relationships exist between animals, which is a common issue in small populations. While imputing, this relatedness positively affects the imputation accuracy from DSN to DSN. Nonetheless, the same results were observed for 30 random unrelated HF animals showing that our findings are robust with regard to the breed under investigation.

The investigation of different phasing strategies for target populations and reference panels showed that phasing significantly affected the imputation accuracy. While Beagle performed best on unphased target populations, the highest imputation accuracy was reached for Minimac when target populations and reference panels were phased with Beagle. In general, better imputation accuracies were observed if the reference panel was phased and when phasing was performed with Beagle.

Although, imputation using Beagle and Minimac was described to produce similar accuracy (Browning and Browning, 2016), we observed an ambiguous performance with Beagle imputing at higher accuracy using bigger reference panels (“1000 bulls” reference panel) while Minimac reached higher accuracy when a small reference panel was available (30 DSN reference panel). Furthermore, using genotype likelihoods as the output of imputation instead of genotypes, which is possible in Minimac, could lead to more precise and higher imputation accuracy. Nonetheless, we believe that the general trends in imputation found in this study should not be affected when using genotype likelihoods. As Beagle does not include an option for estimating genotype likelihoods, we performed all analyses using genotypes.

From literature, the 2-step imputation approach has been suggested to be advantageous in comparison to the 1-step imputation approach with regard to imputation accuracy (VanRaden et al., 2013; van Binsbergen et al., 2014). Our results agree with this when a large amount of intermediate genotypes is available. However, for small population in which this intermediate level is not as abundant our findings suggest the opposite: A higher imputation accuracy was reached in the 1-step imputation approach compared to the 2-step imputation approach. The reduced performance of the 2-step imputation is probably caused by imputation errors from the first imputation step (50 to 700 k) that are propagated into the second imputation step (700 k to sequence level). For example, if around 90% of the genotypes are correctly imputed per imputation step, the accuracy drops after two imputation steps to 81%. Therefore, a 2-step imputation approach is only useful when a large number of animals is available for the first step of imputation and only a small number of sequenced animals for the second step. Furthermore, since the current release of the 1000 Bull Genomes Project provides a large number of sequenced animals that can be used as reference panel (and the number of sequenced animals will increase with time), the 1-step imputation approach should be preferred compared to the 2-step imputation approach.

For a high accuracy of genotype imputation in small populations, several things have to be considered. Consistent with other studies, the imputation accuracy was positively affected if animals of the target population and the reference panels are closer related, e.g., are from the same breed (Ma et al., 2013; Pausch et al., 2017). Thus, imputation accuracy was higher when HF individuals were imputed using a reference panel of other HF animals compared to imputation using animals from various breeds. However, if the number of same breed animals in the reference panel is limited (e.g., in the case of DSN) expanding the reference panel using animals from a related breed such as HF or from various breeds reduced the imputation accuracy. The initial imputation accuracy obtained when using a 30 DSN reference panel was only restored after 17 times more HF animals or 10 times more animals from various breeds were added to the reference panel. In agreement with our results, other studies also showed that the use of a multi-breed instead of a one-breed reference panel increased imputation accuracy (Bouwman and Veerkamp, 2014; Brøndum et al., 2014; Pausch et al., 2017). This is the result of a higher variety of haplotypes segregating in the multi-breed reference panel that could provide a better fit to the linkage disequilibrium existing in the target population. Overall, the best imputation accuracy was reached when a very large multi-breed reference panel such as the “1000 bulls” was used.

While most studies investigating the accuracy of imputation use correlation (Druet et al., 2010; Brøndum et al., 2014; van Binsbergen et al., 2014; Pausch et al., 2017), additional measurements of the accuracy are available and used, among them the calculation of the percentage of correctly imputed genotypes, the relative Manhattan distance (Zhang and Druet, 2010), and other more complex assessments (van Binsbergen et al., 2014). Correlation only describes the linear relationship between two variables, and is not a measurement of distance, which we believe imputation accuracy should be. The easiest way to assess the accuracy of real versus imputed genotypes is the calculation of the percentage of correctly imputed genotypes. However, the calculation of the percentage of correctly imputed genotypes does not distinguish between different types of errors, e.g., if only one allele (observed genotype = 0, imputed genotype = 1) or even two alleles (observed genotype = 0, imputed genotype = 2) at a locus are wrongly imputed. The relative Manhattan distance accounts for these different types of errors and, therefore, should be used when calculating imputation accuracy. Nonetheless, the observations, findings and conclusions of this study were consistent and independent of the measurement used to assess imputation accuracy.

Besides, the overall low imputation accuracy, independent of which of the three measurements for imputation accuracy was used, is caused by the fact that for imputation only variants were used that were polymorphic in either DSN or HF animals. By doing this, around 50% of the variants from the 1000 Bull Genomes Project were not considered, as they are all genotypes that are homozygous to the reference allele for the DSN and HF animals. If those variants would be included into the analysis, the overall imputation accuracy would be artificially inflated as those genotypes would be consistent across the investigated breeds and thus always be correctly imputed.

Mean imputation accuracy with respect to the minor allele frequency (MAF) binned into 10% bins was investigated for all imputations performed. Counterintuitively, imputation accuracies tend to be higher in lower MAF bins, which the authors assume is due to the very limited size of the target population causing imputation accuracy to be overestimated at lower MAFs. Investigation of the different MAF bins on the imputation accuracy as well as the choice of imputation strategy showed that our results and conclusions presented on global imputation accuracy are robust, and replicate in almost all MAF bins. Differences in global imputation accuracy between the different phasing strategies was very small; for the lower MAF bins, the differences are even smaller or not existent (Supplementary Tables 6, 7 – “Phasing of target population and reference panel”). Regarding the choice of imputation software, we observed that Minimac showed slightly better imputation accuracies at higher MAF; Beagle and Minimac perform the same for lower MAF bins (Supplementary Tables 6, 7 – “Comparison of imputation software”), however, this was only observed using the 30 DSN reference panel. Considering 1-step vs. 2-step imputation, the only difference when comparing MAF bins to global imputation accuracy was found for MAF bin (0, 0.1], which showed a slightly lower imputation accuracy for the 2-step imputation approach, all other bins show the same pattern as the global imputation (Supplementary Tables 6, 7 – “Comparison of 1-step and 2-step imputation”).

The estimation of imputation accuracy for HF animals using the data from the 1000 Bull Genomes Project are to a certain degree biased. Since the current version of the 1000 Bull Genomes Project (Run 6) comprises about 25% HF animals, the accuracy of imputation will be better for HF animals than for other cattle breeds using the same “1000 bulls” dataset as a reference panel for imputation.

## Author Contributions

PK, DA, and GB designed the study. PK performed all computational and statistical analysis and drafted the manuscript. PK and DA interpreted the data. DA and GB drafted the manuscript. All authors read and approved the final manuscript.

## Conflict of Interest Statement

The authors declare that the research was conducted in the absence of any commercial or financial relationships that could be construed as a potential conflict of interest.

## Footnotes

1 http://www.1000bullgenomes.com/
