# Supplementary material for: Finding the Optimal Imputation Strategy for Small Cattle Populations
Source: Front Genet. 2019 Feb 18;10:52. doi: 10.3389/fgene.2019.00052 (PMC6387911; doi:10.3389/fgene.2019.00052)
Supplement: Supplementary file 1 [file Data_Sheet_1.docx]

***Supplementary Information***

**Finding the optimal imputation strategy for small cattle populations**

**Paula Korkuć, Danny Arends, Gudrun A. Brockmann**

***Correspondence:** paula.korkuc@hu-berlin.de

**Supplementary Table 1:** Significance of phasing strategies of the target populations and reference panels. ANOVA p-values were calculated separately for each target population (DSN, HF), imputation software (Beagle, Minimac) and composition of the reference panel (30 DSN or 30 HF, and “1000 bulls”) based on Manhattan distance. P values <0.05 were considered significant and are highlighted in bold.

| **Target population** | **Imputation software** | **Reference panel** | **ANOVA p-value for target population phasing** | **ANOVA p-value for reference panel phasing** |
| --- | --- | --- | --- | --- |
| DSN | Beagle | 30 DSN | 9.1E-01 | 6.0E-01 |
|  |  | 1000 bulls | **1.0E-07** | 1.2E-01 |
|  | Minimac | 30 DSN | **8.7E-10** | **1.2E-03** |
|  |  | 1000 bulls | **1.5E-24** | **3.1E-31** |
| HF | Beagle | 30 HF | 8.5E-01 | 5.4E-01 |
|  |  | 1000 bulls | **1.8E-22** | **2.1E-02** |
|  | Minimac | 30 HF | **8.2E-08** | **6.3E-03** |
|  |  | 1000 bulls | **5.8E-26** | **1.7E-34** |

**Supplementary Table 2**: Mean imputation accuracy with regard to different phasing strategies. The imputation was performed from 50k to sequence level using different imputation software, target populations, reference panels for imputation, and phasing of the target population and reference panel. The imputation accuracy was calculated using either relative Manhattan distance (d_M_), percent identity or correlation (r) between observed and imputed genotypes. Also the pairwise correlation coefficients between the three accuracy measurements are provided.

| **Imputation software** | **Target population** | **Reference panel** | **Phasing of target population** | **Phasing of reference panel** | **Mean d_M_** | **Mean percent identity** | **Mean r** | **r  (d_M_, percent identity)** | **r  (d_M_, r)** | **r (percent identity, r)** |
| --- | --- | --- | --- | --- | --- | --- | --- | --- | --- | --- |
| Beagle | DSN | 30 DSN | Unphased | Beagle | 0.908 | 0.837 | 0.745 | 0.882 | 0.905 | 0.635 |
|  |  |  |  | Eagle | 0.908 | 0.837 | 0.745 | 0.881 | 0.901 | 0.625 |
|  |  |  | Eagle | Beagle | 0.908 | 0.837 | 0.745 | 0.881 | 0.904 | 0.631 |
|  |  |  |  | Eagle | 0.908 | 0.837 | 0.745 | 0.881 | 0.901 | 0.625 |
|  |  |  | Beagle | Beagle | 0.908 | 0.837 | 0.746 | 0.881 | 0.902 | 0.628 |
|  |  |  |  | Eagle | 0.908 | 0.838 | 0.746 | 0.879 | 0.898 | 0.618 |
|  |  | 1000 bulls | Unphased | Beagle | 0.940 | 0.889 | 0.836 | 0.907 | 0.903 | 0.676 |
|  |  |  |  | Eagle | 0.939 | 0.888 | 0.834 | 0.899 | 0.889 | 0.640 |
|  |  |  | Eagle | Beagle | 0.937 | 0.883 | 0.828 | 0.901 | 0.890 | 0.654 |
|  |  |  |  | Eagle | 0.936 | 0.883 | 0.826 | 0.893 | 0.887 | 0.636 |
|  |  |  | Beagle | Beagle | 0.937 | 0.884 | 0.829 | 0.899 | 0.890 | 0.645 |
|  |  |  |  | Eagle | 0.936 | 0.883 | 0.826 | 0.891 | 0.877 | 0.613 |
|  | HF | 30 HF | Unphased | Beagle | 0.913 | 0.846 | 0.767 | 0.893 | 0.953 | 0.727 |
|  |  |  |  | Eagle | 0.914 | 0.847 | 0.767 | 0.895 | 0.952 | 0.726 |
|  |  |  | Eagle | Beagle | 0.913 | 0.846 | 0.767 | 0.893 | 0.953 | 0.727 |
|  |  |  |  | Eagle | 0.914 | 0.847 | 0.767 | 0.895 | 0.952 | 0.726 |
|  |  |  | Beagle | Beagle | 0.914 | 0.847 | 0.768 | 0.896 | 0.959 | 0.743 |
|  |  |  |  | Eagle | 0.914 | 0.848 | 0.768 | 0.896 | 0.956 | 0.739 |
|  |  | 1000 bulls | Unphased | Beagle | 0.960 | 0.926 | 0.898 | 0.966 | 0.956 | 0.864 |
|  |  |  |  | Eagle | 0.959 | 0.923 | 0.894 | 0.968 | 0.953 | 0.865 |
|  |  |  | Eagle | Beagle | 0.952 | 0.911 | 0.877 | 0.976 | 0.964 | 0.902 |
|  |  |  |  | Eagle | 0.951 | 0.908 | 0.873 | 0.978 | 0.968 | 0.911 |
|  |  |  | Beagle | Beagle | 0.953 | 0.913 | 0.880 | 0.967 | 0.961 | 0.880 |
|  |  |  |  | Eagle | 0.952 | 0.909 | 0.875 | 0.967 | 0.958 | 0.878 |
| Minimac | DSN | 30 DSN | Unphased | Beagle | 0.913 | 0.847 | 0.751 | 0.657 | 0.526 | -0.195 |
|  |  |  |  | Eagle | 0.912 | 0.846 | 0.747 | 0.656 | 0.499 | -0.223 |
|  |  |  | Eagle | Beagle | 0.915 | 0.851 | 0.756 | 0.636 | 0.571 | -0.176 |
|  |  |  |  | Eagle | 0.914 | 0.849 | 0.752 | 0.639 | 0.541 | -0.204 |
|  |  |  | Beagle | Beagle | 0.915 | 0.851 | 0.757 | 0.612 | 0.544 | -0.233 |
|  |  |  |  | Eagle | 0.914 | 0.850 | 0.753 | 0.618 | 0.514 | -0.257 |
|  |  | 1000 bulls | Unphased | Beagle | 0.925 | 0.864 | 0.791 | 0.762 | 0.641 | 0.084 |
|  |  |  |  | Eagle | 0.921 | 0.859 | 0.779 | 0.741 | 0.606 | 0.012 |
|  |  |  | Eagle | Beagle | 0.929 | 0.872 | 0.803 | 0.729 | 0.694 | 0.089 |
|  |  |  |  | Eagle | 0.924 | 0.865 | 0.787 | 0.698 | 0.645 | -0.012 |
|  |  |  | Beagle | Beagle | 0.930 | 0.874 | 0.805 | 0.692 | 0.662 | -0.002 |
|  |  |  |  | Eagle | 0.924 | 0.865 | 0.788 | 0.691 | 0.623 | -0.046 |
|  | HF | 30 HF | Unphased | Beagle | 0.913 | 0.849 | 0.752 | 0.651 | 0.619 | -0.142 |
|  |  |  |  | Eagle | 0.913 | 0.848 | 0.748 | 0.646 | 0.621 | -0.150 |
|  |  |  | Eagle | Beagle | 0.915 | 0.853 | 0.757 | 0.620 | 0.647 | -0.148 |
|  |  |  |  | Eagle | 0.914 | 0.851 | 0.752 | 0.620 | 0.648 | -0.148 |
|  |  |  | Beagle | Beagle | 0.916 | 0.854 | 0.758 | 0.575 | 0.680 | -0.161 |
|  |  |  |  | Eagle | 0.915 | 0.852 | 0.754 | 0.584 | 0.655 | -0.184 |
|  |  | 1000 bulls | Unphased | Beagle | 0.932 | 0.876 | 0.819 | 0.894 | 0.815 | 0.525 |
|  |  |  |  | Eagle | 0.927 | 0.869 | 0.802 | 0.857 | 0.760 | 0.374 |
|  |  |  | Eagle | Beagle | 0.939 | 0.888 | 0.835 | 0.887 | 0.876 | 0.592 |
|  |  |  |  | Eagle | 0.931 | 0.877 | 0.812 | 0.841 | 0.802 | 0.398 |
|  |  |  | Beagle | Beagle | 0.940 | 0.891 | 0.838 | 0.876 | 0.877 | 0.572 |
|  |  |  |  | Eagle | 0.932 | 0.878 | 0.814 | 0.816 | 0.806 | 0.361 |


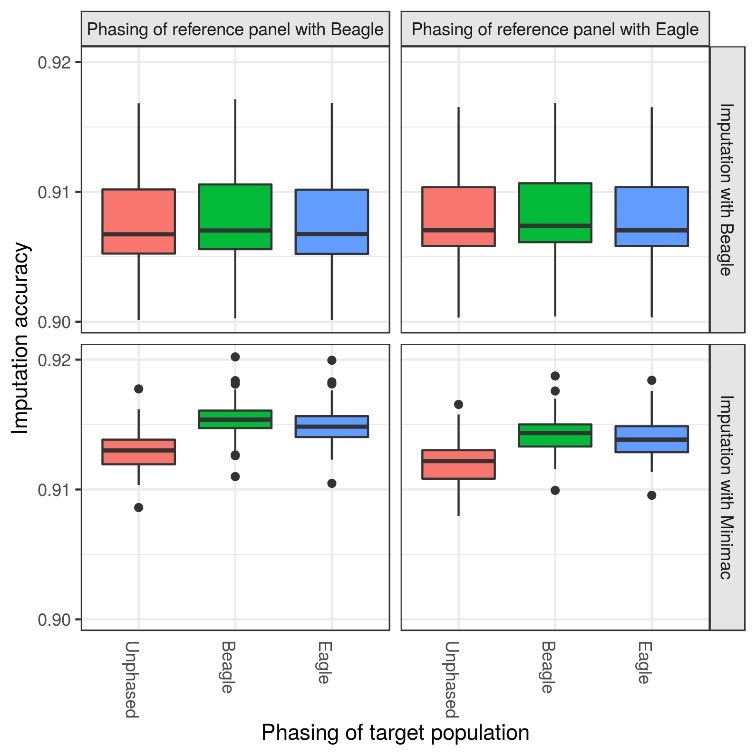


**Supplementary Figure 1:** Comparison of different phasing strategies using the DSN target population and the 30 DSN reference panel. The target population was either unphased (red), phased with Beagle (blue) or phased with Eagle (green), while the reference panel was phased with Beagle (left panel) or Eagle (right panel). Imputation was performed using Beagle (top panel) or Minimac (bottom panel) from 50k to sequence level. Imputation accuracy was calculated using relative Manhattan distance. When using Beagle for imputation, no differences in imputation accuracy were observed for the different phasing strategies. In contrast, when Minimac was used for imputation, the imputation accuracy was highest for target populations and reference panels both phased with Beagle.


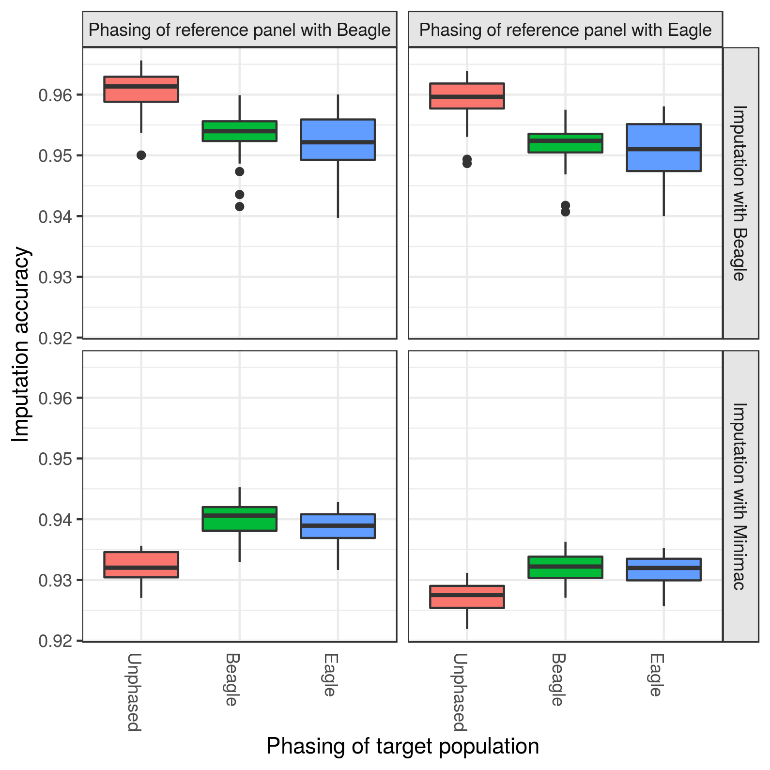


**Supplementary Figure 2:** Comparison of different phasing strategies using the HF target population and the “1000 bulls” reference panel. The target population was either unphased (red), phased with Beagle (blue) or phased with Eagle (green), while the reference panel was phased with Beagle (left panel) or Eagle (right panel). Imputation was performed using Beagle (top panel) or Minimac (bottom panel) from 50k to sequence level. Imputation accuracy was calculated using relative Manhattan distance. The same results were observed as for the DSN target population using the “1000 bulls” reference panel (Figure 2).


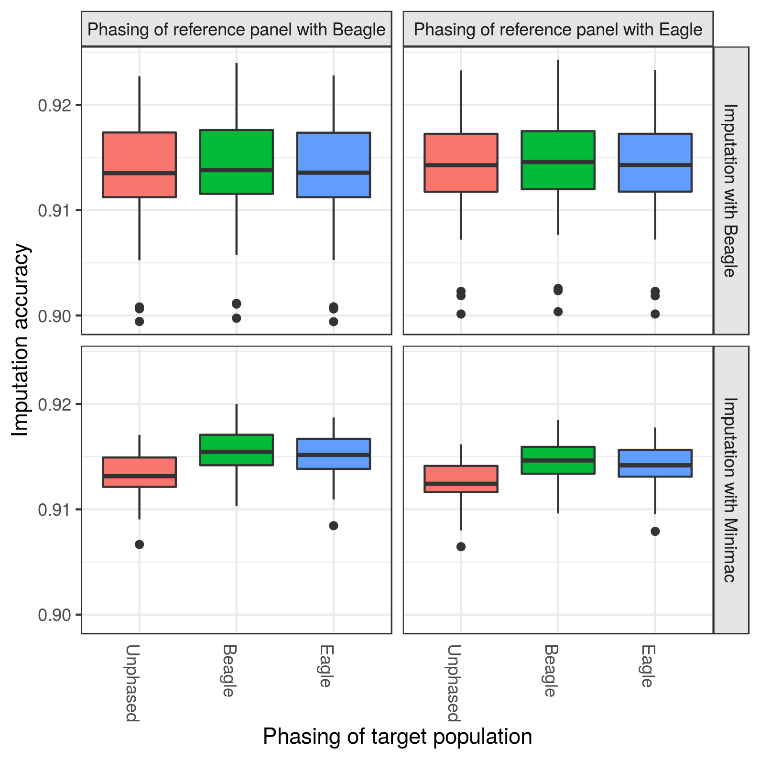


**Supplementary Figure 3:** Comparison of different phasing strategies using the HF target population and the 30 HF reference panel. The target population was either unphased (red), phased with Beagle (blue) or phased with Eagle (green), while the reference panel was phased with Beagle (left panel) or Eagle (right panel). Imputation was performed using Beagle (top panel) or Minimac (bottom panel) from 50k to sequence level. Imputation accuracy was calculated using relative Manhattan distance. The same results were observed as for the DSN target population using the 30 DSN reference panel (Supplementary Figure 1).

**Supplementary Table 3:** Mean imputation accuracy with regard to different imputation software tools. The imputation was performed from 50k to sequence level with either Minimac or Beagle using differently sized and composed reference panels for imputation. The target populations and reference panels were phased using Beagle. The imputation accuracy was calculated using either relative Manhattan distance (dM), percent identity or correlation (r) between observed and imputed genotypes. Also the pairwise correlation coefficients between the three accuracy measurements are provided.

| **Target population** | **Imputation software** | **Reference panel** | **Mean d_M_** | **Mean percent identity** | **Mean r** | **r  (d_M_, percent identity)** | **r  (d_M_, r)** | **r  (percent identity, r)** |
| --- | --- | --- | --- | --- | --- | --- | --- | --- |
| DSN | Beagle | 30 DSN | 0.906 | 0.834 | 0.737 | 0.917 | 0.971 | 0.805 |
|  |  | 1000 bulls | 0.932 | 0.876 | 0.811 | 0.916 | 0.883 | 0.666 |
|  | Minimac | 30 DSN | 0.910 | 0.842 | 0.734 | 0.768 | 0.704 | 0.154 |
|  |  | 1000 bulls | 0.924 | 0.864 | 0.786 | 0.811 | 0.759 | 0.298 |
| HF | Beagle | 30 HF | 0.911 | 0.843 | 0.752 | 0.951 | 0.969 | 0.857 |
|  |  | 1000 bulls | 0.947 | 0.902 | 0.858 | 0.986 | 0.986 | 0.956 |
|  | Minimac | 30 HF | 0.911 | 0.847 | 0.734 | 0.681 | 0.796 | 0.141 |
|  |  | 1000 bulls | 0.934 | 0.882 | 0.815 | 0.931 | 0.938 | 0.774 |


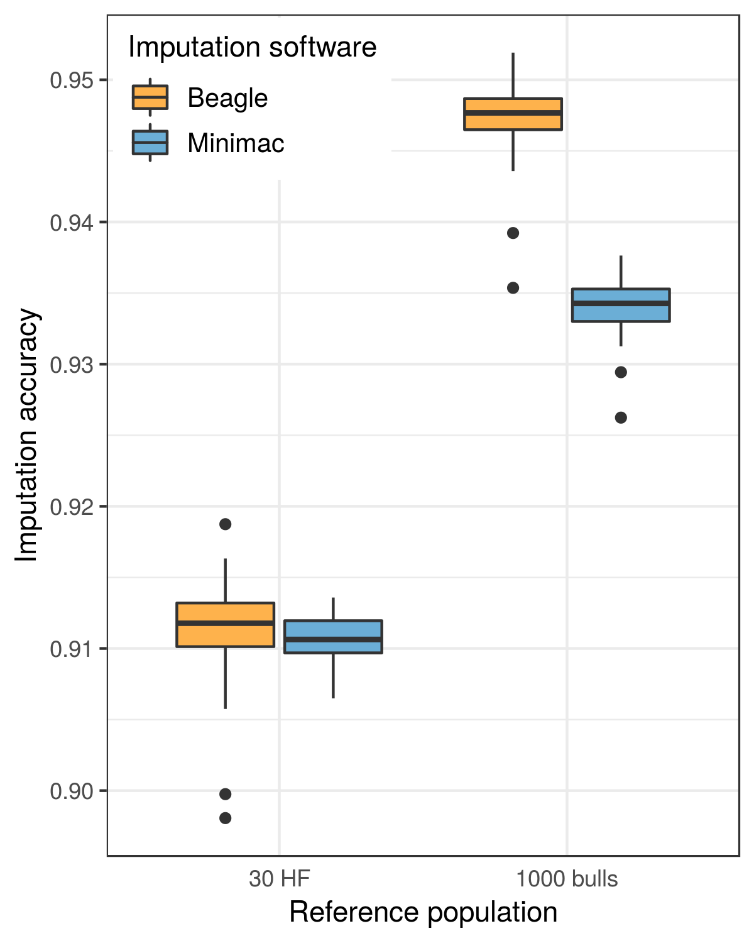


**Supplementary Figure 4:** Imputation software comparison for the imputation of the HF target population. Either the 30 HF or the “1000 bulls” reference panel was used for imputation from 50k to sequence level. Imputation accuracy was calculated using relative Manhattan distance. We observed a significantly higher imputation accuracy with Beagle (orange) than with Minimac (blue) using the “1000 bulls” reference panel (t-test p-value=8.6E-22). The accuracy of imputation with the smaller reference panel (30 HF) showed no significant difference between the imputation tools Beagle and Minimac.

**Supplementary Table 4:** Mean imputation accuracy with regard to different imputation approaches. The imputation was performed using a 1-step or 2-step approach with Beagle using differently sized and composed reference panels for imputation. The target populations and reference panels were phased using Beagle. The imputation accuracy was calculated using either relative Manhattan distance (d_M_), percent identity or correlation (r) between observed and imputed genotypes. Also the pairwise correlation coefficients between the three accuracy measurements are provided.

| **Target population** | **Imputation strategy** | **Reference panel (50k to 700k)** | **Reference panel (to sequence level)** | **Mean d_M_** | **Mean percent identity** | **Mean r** | **r  (d_M_, percent identity)** | **r  (d_M_, r)** | **r  (percent identity, r)** |
| --- | --- | --- | --- | --- | --- | --- | --- | --- | --- |
| DSN | 1-step | - | 30 DSN | 0.906 | 0.834 | 0.737 | 0.917 | 0.971 | 0.805 |
|  |  |  | 1000 bulls | 0.932 | 0.876 | 0.811 | 0.916 | 0.883 | 0.666 |
|  | 2-step | 30 DSN | 30 DSN | 0.896 | 0.820 | 0.709 | 0.918 | 0.975 | 0.817 |
|  |  |  | 1000 bulls | 0.897 | 0.822 | 0.712 | 0.923 | 0.977 | 0.830 |
|  |  | 1000 bulls | 30 DSN | 0.914 | 0.847 | 0.759 | 0.881 | 0.938 | 0.688 |
|  |  |  | 1000 bulls | 0.921 | 0.857 | 0.778 | 0.863 | 0.860 | 0.540 |
| HF | 1-step | - | 30 HF | 0.911 | 0.843 | 0.752 | 0.951 | 0.969 | 0.857 |
|  |  |  | 1000 bulls | 0.947 | 0.902 | 0.858 | 0.986 | 0.986 | 0.956 |
|  | 2-step | 30 HF | 30 HF | 0.901 | 0.829 | 0.725 | 0.952 | 0.974 | 0.868 |
|  |  |  | 1000 bulls | 0.904 | 0.834 | 0.734 | 0.955 | 0.976 | 0.877 |
|  |  | 1000 bulls | 30 HF | 0.925 | 0.867 | 0.794 | 0.975 | 0.981 | 0.929 |
|  |  |  | 1000 bulls | 0.938 | 0.887 | 0.831 | 0.985 | 0.987 | 0.959 |


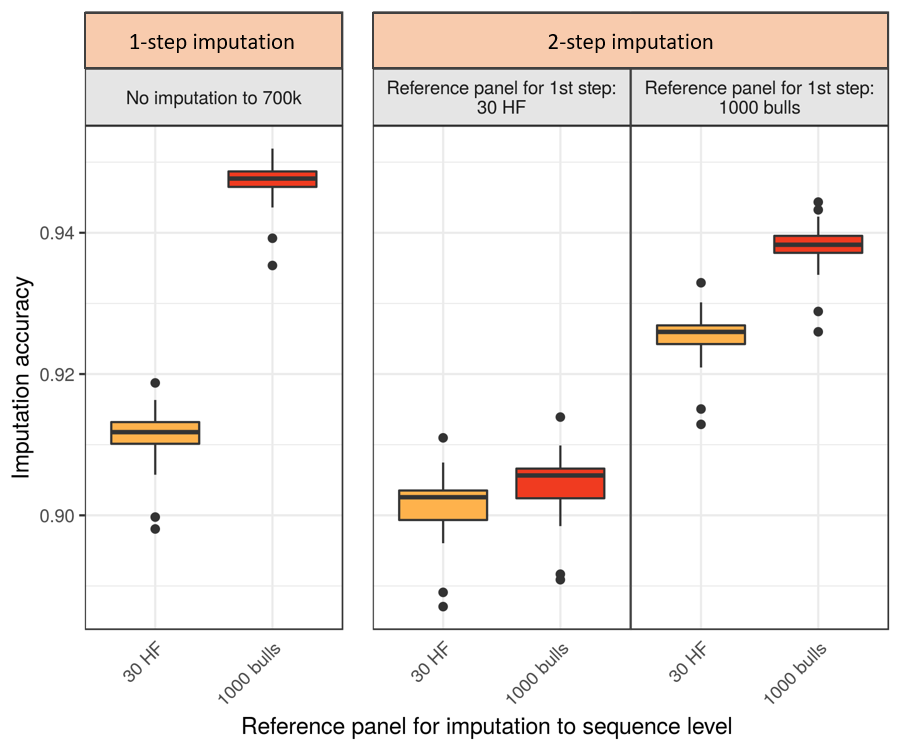


**Supplementary Figure 5:** Comparison of imputation accuracy between the 1-step and 2-step imputation approach using Beagle for the HF target population. Two reference panels (30 HF and “1000 bulls”) were used for the 1-step imputation from 50k to sequence level. The same two panels were used for the first (50k to 700k) and second step (from 700k to sequence level) in the 2-step imputation approach. The colors refer to the reference panels used for the imputation to sequence level (yellow – 30 HF reference panel, orange – “1000 bulls” reference panel). Imputation accuracy was calculated using relative Manhattan distance. The same results were observed as for the DSN target population (Figure 4).

**Supplementary Table 5:** Mean imputation accuracy with regard to different reference panels. The imputation was performed from 50k to sequence level with Beagle. To the initial reference panel consisting of 30 DSN or 30 HF additional 100 HF or 100 animals from various breeds (VAR) were added successively. The target populations and reference panels were phased using Beagle. The imputation accuracy was calculated using either relative Manhattan distance (d_M_), percent identity or correlation (r) between observed and imputed genotypes. Also the pairwise correlation coefficients between the three accuracy measurements are provided.

| **Target population** | **Reference panel** | **Mean d_M_** | **Mean percent identity** | **Mean r** | **r  (d_M_, percent identity)** | **r  (d_M_, r)** | **r  (percent identity, r)** |
| --- | --- | --- | --- | --- | --- | --- | --- |
| DSN | 30 DSN | 0.906 | 0.834 | 0.737 | 0.917 | 0.971 | 0.805 |
|  | 30 DSN + 100 HF | 0.899 | 0.824 | 0.706 | 0.808 | 0.857 | 0.429 |
|  | 30 DSN + 200 HF | 0.898 | 0.824 | 0.702 | 0.796 | 0.812 | 0.346 |
|  | 30 DSN + 300 HF | 0.900 | 0.827 | 0.709 | 0.790 | 0.814 | 0.341 |
|  | 30 DSN + 400 HF | 0.903 | 0.831 | 0.717 | 0.793 | 0.817 | 0.351 |
|  | 30 DSN + 500 HF | 0.906 | 0.835 | 0.726 | 0.798 | 0.838 | 0.391 |
|  | 30 DSN + 100 VAR | 0.902 | 0.828 | 0.715 | 0.774 | 0.757 | 0.224 |
|  | 30 DSN + 200 VAR | 0.902 | 0.828 | 0.715 | 0.743 | 0.660 | 0.050 |
|  | 30 DSN + 300 VAR | 0.904 | 0.832 | 0.721 | 0.734 | 0.679 | 0.063 |
|  | 30 DSN + 400 VAR | 0.906 | 0.835 | 0.729 | 0.744 | 0.712 | 0.125 |
|  | 30 DSN + 500 VAR | 0.909 | 0.839 | 0.737 | 0.775 | 0.718 | 0.182 |
| HF | 30 HF | 0.919 | 0.855 | 0.752 | 0.827 | 0.774 | 0.348 |
|  | 30 HF + 100 HF | 0.931 | 0.873 | 0.757 | 0.904 | 0.862 | 0.613 |
|  | 30 HF + 200 HF | 0.911 | 0.843 | 0.762 | 0.951 | 0.969 | 0.857 |
|  | 30 HF + 300 HF | 0.913 | 0.845 | 0.771 | 0.943 | 0.963 | 0.833 |
|  | 30 HF + 400 HF | 0.914 | 0.848 | 0.781 | 0.947 | 0.965 | 0.843 |
|  | 30 HF + 500 HF | 0.917 | 0.852 | 0.791 | 0.949 | 0.967 | 0.850 |
|  | 30 HF + 100 VAR | 0.920 | 0.857 | 0.732 | 0.955 | 0.970 | 0.866 |
|  | 30 HF + 200 VAR | 0.924 | 0.863 | 0.732 | 0.961 | 0.973 | 0.882 |
|  | 30 HF + 300 VAR | 0.907 | 0.838 | 0.738 | 0.869 | 0.867 | 0.552 |
|  | 30 HF + 400 VAR | 0.907 | 0.839 | 0.750 | 0.841 | 0.828 | 0.443 |
|  | 30 HF + 500 VAR | 0.909 | 0.842 | 0.759 | 0.823 | 0.800 | 0.370 |


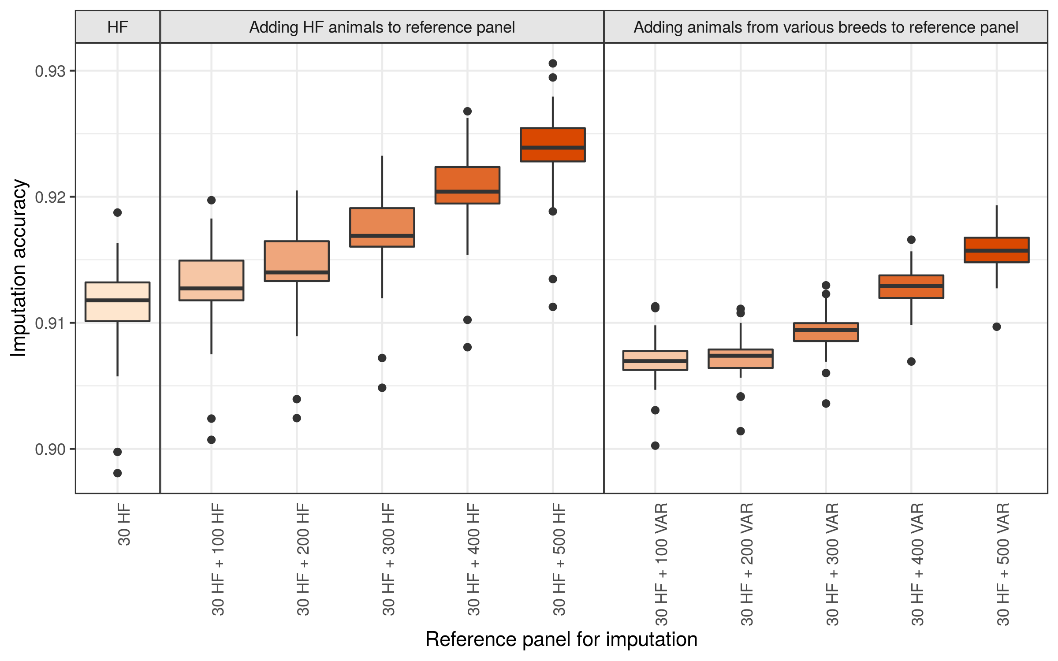


**Supplementary Figure 6:** Comparison of imputation accuracy with regard to differently sized and composed reference panels for the HF target population. The imputation was performed from 50k to sequence level with Beagle using the 30 HF reference panel and successively adding 100 HF animals or animals from various breeds (VAR) to the initial 30 HF reference panel. The imputation accuracy was calculated as relative Manhattan distance. The intensity of the color refers to the total number of animals in the reference panel. The same results were observed as for the DSN target population (Figure 5).

**Supplementary Table 6:** Mean imputation accuracy with respect to the minor allele frequency of the sequenced DSN or HF animals. Imputation was performed for the different imputation setups including the comparison of 1) phasing strategies for the target population (unphased, phased with Beagle, phased with Eagle) and reference panel (phased with Beagle, phased with Eagle), 2) imputation software (Beagle and Minimac), 3) 1-step and 2-step imputation, and 4) adding successively HF animals or animals from various breeds (VAR) to the reference panel. The imputation accuracy was calculated using relative Manhattan distance (d_M_) between observed and imputed genotypes for the minor allele frequency bins (0, 0.1], (0.1, 0.2] ,(0.2, 0.3] ,(0.3, 0.4] ,(0.4, 0.5].

| **1) Phasing of target population and reference panel** | | | | | | | | | | | |
| --- | --- | --- | --- | --- | --- | --- | --- | --- | --- | --- | --- |
| **Target population** | **Phasing of**  **target population** | **Phasing of**  **reference panel** | **Imputation software** | **Reference panel**  **(50k to 700k)** | **Reference panel**  **(to sequence level)** | **Mean d_M_**  **MAF bin (0,0.1]** | **Mean d_M_**  **MAF bin (0.1,0.2]** | **Mean d_M_**  **MAF bin (0.2,0.3]** | **Mean d_M_**  **MAF bin (0.3,0.4]** | **Mean d_M_**  **MAF bin (0.4,0.5]** | **Mean d_M_**  **overall** |
| DSN | Beagle | Beagle | Beagle | - | 30 DSN | 0.955 | 0.862 | 0.792 | 0.746 | 0.715 | 0.908 |
|  |  |  |  | - | 1000 bulls | 0.961 | 0.894 | 0.863 | 0.841 | 0.835 | 0.937 |
|  |  |  | Minimac | - | 30 DSN | 0.954 | 0.870 | 0.808 | 0.775 | 0.755 | 0.915 |
|  |  |  |  | - | 1000 bulls | 0.959 | 0.885 | 0.845 | 0.820 | 0.805 | 0.930 |
|  |  | Eagle | Beagle | - | 30 DSN | 0.955 | 0.862 | 0.791 | 0.746 | 0.714 | 0.908 |
|  |  |  |  | - | 1000 bulls | 0.960 | 0.893 | 0.859 | 0.838 | 0.830 | 0.936 |
|  |  |  | Minimac | - | 30 DSN | 0.954 | 0.868 | 0.805 | 0.768 | 0.747 | 0.914 |
|  |  |  |  | - | 1000 bulls | 0.957 | 0.878 | 0.832 | 0.801 | 0.782 | 0.924 |
|  | Eagle | Beagle | Beagle | - | 30 DSN | 0.955 | 0.861 | 0.792 | 0.745 | 0.713 | 0.908 |
|  |  |  |  | - | 1000 bulls | 0.960 | 0.894 | 0.861 | 0.841 | 0.833 | 0.937 |
|  |  |  | Minimac | - | 30 DSN | 0.954 | 0.869 | 0.807 | 0.774 | 0.753 | 0.915 |
|  |  |  |  | - | 1000 bulls | 0.958 | 0.884 | 0.843 | 0.818 | 0.802 | 0.929 |
|  |  | Eagle | Beagle | - | 30 DSN | 0.955 | 0.862 | 0.791 | 0.745 | 0.712 | 0.908 |
|  |  |  |  | - | 1000 bulls | 0.960 | 0.893 | 0.859 | 0.838 | 0.829 | 0.936 |
|  |  |  | Minimac | - | 30 DSN | 0.954 | 0.868 | 0.804 | 0.767 | 0.745 | 0.914 |
|  |  |  |  | - | 1000 bulls | 0.957 | 0.878 | 0.831 | 0.801 | 0.781 | 0.924 |
|  | Unphased | Beagle | Beagle | - | 30 DSN | 0.955 | 0.861 | 0.792 | 0.745 | 0.713 | 0.908 |
|  |  |  |  | - | 1000 bulls | 0.961 | 0.899 | 0.870 | 0.850 | 0.843 | 0.940 |
|  |  |  | Minimac | - | 30 DSN | 0.954 | 0.867 | 0.803 | 0.767 | 0.746 | 0.913 |
|  |  |  |  | - | 1000 bulls | 0.956 | 0.878 | 0.832 | 0.805 | 0.789 | 0.925 |
|  |  | Eagle | Beagle | - | 30 DSN | 0.955 | 0.862 | 0.791 | 0.745 | 0.712 | 0.908 |
|  |  |  |  | - | 1000 bulls | 0.961 | 0.898 | 0.867 | 0.847 | 0.839 | 0.939 |
|  |  |  | Minimac | - | 30 DSN | 0.954 | 0.866 | 0.800 | 0.761 | 0.739 | 0.912 |
|  |  |  |  | - | 1000 bulls | 0.956 | 0.874 | 0.823 | 0.791 | 0.770 | 0.921 |
| HF | Beagle | Beagle | Beagle | - | 30 HF | 0.956 | 0.867 | 0.804 | 0.749 | 0.723 | 0.914 |
|  |  |  |  | - | 1000 bulls | 0.965 | 0.919 | 0.898 | 0.883 | 0.886 | 0.953 |
|  |  |  | Minimac | - | 30 HF | 0.953 | 0.866 | 0.808 | 0.765 | 0.745 | 0.916 |
|  |  |  |  | - | 1000 bulls | 0.961 | 0.899 | 0.865 | 0.841 | 0.834 | 0.940 |
|  |  | Eagle | Beagle | - | 30 HF | 0.956 | 0.867 | 0.804 | 0.749 | 0.725 | 0.914 |
|  |  |  |  | - | 1000 bulls | 0.965 | 0.916 | 0.894 | 0.879 | 0.881 | 0.952 |
|  |  |  | Minimac | - | 30 HF | 0.953 | 0.865 | 0.804 | 0.758 | 0.736 | 0.915 |
|  |  |  |  | - | 1000 bulls | 0.959 | 0.889 | 0.845 | 0.814 | 0.802 | 0.932 |
|  | Eagle | Beagle | Beagle | - | 30 HF | 0.956 | 0.866 | 0.803 | 0.747 | 0.720 | 0.913 |
|  |  |  |  | - | 1000 bulls | 0.965 | 0.917 | 0.895 | 0.879 | 0.882 | 0.952 |
|  |  |  | Minimac | - | 30 HF | 0.953 | 0.866 | 0.806 | 0.762 | 0.742 | 0.915 |
|  |  |  |  | - | 1000 bulls | 0.961 | 0.897 | 0.861 | 0.836 | 0.830 | 0.939 |
|  |  | Eagle | Beagle | - | 30 HF | 0.956 | 0.866 | 0.803 | 0.747 | 0.722 | 0.914 |
|  |  |  |  | - | 1000 bulls | 0.965 | 0.916 | 0.893 | 0.877 | 0.878 | 0.951 |
|  |  |  | Minimac | - | 30 HF | 0.953 | 0.864 | 0.803 | 0.756 | 0.734 | 0.914 |
|  |  |  |  | - | 1000 bulls | 0.959 | 0.888 | 0.843 | 0.811 | 0.799 | 0.931 |
|  | Unphased | Beagle | Beagle | - | 30 HF | 0.956 | 0.866 | 0.803 | 0.747 | 0.720 | 0.913 |
|  |  |  |  | - | 1000 bulls | 0.969 | 0.931 | 0.915 | 0.902 | 0.906 | 0.960 |
|  |  |  | Minimac | - | 30 HF | 0.953 | 0.862 | 0.802 | 0.756 | 0.735 | 0.913 |
|  |  |  |  | - | 1000 bulls | 0.958 | 0.887 | 0.847 | 0.818 | 0.809 | 0.932 |
|  |  | Eagle | Beagle | - | 30 HF | 0.956 | 0.866 | 0.803 | 0.747 | 0.722 | 0.914 |
|  |  |  |  | - | 1000 bulls | 0.968 | 0.929 | 0.913 | 0.900 | 0.903 | 0.959 |
|  |  |  | Minimac | - | 30 HF | 0.953 | 0.861 | 0.800 | 0.751 | 0.727 | 0.913 |
|  |  |  |  | - | 1000 bulls | 0.958 | 0.881 | 0.833 | 0.799 | 0.784 | 0.927 |
| **2) Comparison of imputation software (Beagle versus Minimac)** | | | | | | | | | | | |
| **Target population** | **Phasing of**  **target population** | **Phasing of**  **reference panel** | **Imputation software** | **Reference panel**  **(50k to 700k)** | **Reference panel**  **(to sequence level)** | **Mean d_M_**  **MAF bin (0,0.1]** | **Mean d_M_**  **MAF bin (0.1,0.2]** | **Mean d_M_**  **MAF bin (0.2,0.3]** | **Mean d_M_**  **MAF bin (0.3,0.4]** | **Mean d_M_**  **MAF bin (0.4,0.5]** | **Mean d_M_**  **overall** |
| DSN | Beagle | Beagle | Beagle | - | 30 DSN | 0.954 | 0.862 | 0.793 | 0.739 | 0.707 | 0.906 |
|  |  |  |  | - | 1000 bulls | 0.959 | 0.890 | 0.851 | 0.826 | 0.817 | 0.932 |
|  |  |  | Minimac | - | 30 DSN | 0.953 | 0.862 | 0.799 | 0.756 | 0.734 | 0.910 |
|  |  |  |  | - | 1000 bulls | 0.957 | 0.880 | 0.834 | 0.801 | 0.786 | 0.924 |
| HF | Beagle | Beagle | Beagle | - | 30 HF | 0.956 | 0.863 | 0.799 | 0.747 | 0.722 | 0.911 |
|  |  |  |  | - | 1000 bulls | 0.964 | 0.908 | 0.884 | 0.868 | 0.867 | 0.947 |
|  |  |  | Minimac | - | 30 HF | 0.954 | 0.860 | 0.797 | 0.752 | 0.733 | 0.911 |
|  |  |  |  | - | 1000 bulls | 0.960 | 0.890 | 0.852 | 0.826 | 0.818 | 0.934 |
| **3) Comparison of 1-step and 2-step imputation** | | | | | | | | | | | |
| **Target population** | **Phasing of**  **target population** | **Phasing of**  **reference panel** | **Imputation software** | **Reference panel**  **(50k to 700k)** | **Reference panel**  **(to sequence level)** | **Mean d_M_**  **MAF bin (0,0.1]** | **Mean d_M_**  **MAF bin (0.1,0.2]** | **Mean d_M_**  **MAF bin (0.2,0.3]** | **Mean d_M_**  **MAF bin (0.3,0.4]** | **Mean d_M_**  **MAF bin (0.4,0.5]** | **Mean d_M_**  **overall** |
| DSN | Beagle | Beagle | Beagle | - | 30 DSN | 0.954 | 0.862 | 0.793 | 0.739 | 0.707 | 0.906 |
|  |  |  |  | - | 1000 bulls | 0.959 | 0.890 | 0.851 | 0.826 | 0.817 | 0.932 |
|  |  |  |  | 30 DSN | 30 DSN | 0.951 | 0.849 | 0.771 | 0.705 | 0.670 | 0.896 |
|  |  |  |  | 30 DSN | 1000 bulls | 0.944 | 0.842 | 0.777 | 0.730 | 0.702 | 0.897 |
|  |  |  |  | 1000 bulls | 30 DSN | 0.952 | 0.864 | 0.810 | 0.774 | 0.759 | 0.914 |
|  |  |  |  | 1000 bulls | 1000 bulls | 0.946 | 0.874 | 0.835 | 0.810 | 0.799 | 0.921 |
| HF | Beagle | Beagle | Beagle | - | 30 HF | 0.956 | 0.863 | 0.799 | 0.747 | 0.722 | 0.911 |
|  |  |  |  | - | 1000 bulls | 0.964 | 0.908 | 0.884 | 0.868 | 0.867 | 0.947 |
|  |  |  |  | 30 HF | 30 HF | 0.954 | 0.851 | 0.774 | 0.711 | 0.690 | 0.901 |
|  |  |  |  | 30 HF | 1000 bulls | 0.948 | 0.846 | 0.784 | 0.739 | 0.723 | 0.904 |
|  |  |  |  | 1000 bulls | 30 HF | 0.956 | 0.875 | 0.832 | 0.802 | 0.798 | 0.925 |
|  |  |  |  | 1000 bulls | 1000 bulls | 0.956 | 0.896 | 0.870 | 0.850 | 0.849 | 0.938 |
|  |  |  |  |  |  |  |  |  |  |  |  |
|  |  |  |  |  |  |  |  |  |  |  |  |
|  |  |  |  |  |  |  |  |  |  |  |  |
|  |  |  |  |  |  |  |  |  |  |  |  |
|  |  |  |  |  |  |  |  |  |  |  |  |
|  |  |  |  |  |  |  |  |  |  |  |  |
| **4) Adding HF animals or animals from various breeds to the reference panel** | | | | | | | | | | | |
| **Target population** | **Phasing of**  **target population** | **Phasing of**  **reference panel** | **Imputation software** | **Reference panel**  **(50k to 700k)** | **Reference panel**  **(to sequence level)** | **Mean d_M_**  **MAF bin (0,0.1]** | **Mean d_M_**  **MAF bin (0.1,0.2]** | **Mean d_M_**  **MAF bin (0.2,0.3]** | **Mean d_M_**  **MAF bin (0.3,0.4]** | **Mean d_M_**  **MAF bin (0.4,0.5]** | **Mean d_M_**  **overall** |
| DSN | Beagle | Beagle | Beagle | - | 30 DSN | 0.954 | 0.862 | 0.793 | 0.739 | 0.707 | 0.906 |
|  |  |  |  | - | 30 DSN + 100 HF | 0.951 | 0.853 | 0.775 | 0.714 | 0.685 | 0.899 |
|  |  |  |  | - | 30 DSN + 200 HF | 0.951 | 0.850 | 0.772 | 0.715 | 0.688 | 0.898 |
|  |  |  |  | - | 30 DSN + 300 HF | 0.951 | 0.852 | 0.777 | 0.723 | 0.699 | 0.900 |
|  |  |  |  | - | 30 DSN + 400 HF | 0.951 | 0.854 | 0.783 | 0.731 | 0.710 | 0.903 |
|  |  |  |  | - | 30 DSN + 500 HF | 0.951 | 0.857 | 0.790 | 0.741 | 0.720 | 0.906 |
|  |  |  |  | - | 30 DSN + 100 VAR | 0.953 | 0.858 | 0.784 | 0.722 | 0.688 | 0.902 |
|  |  |  |  | - | 30 DSN + 200 VAR | 0.953 | 0.857 | 0.784 | 0.724 | 0.691 | 0.902 |
|  |  |  |  | - | 30 DSN + 300 VAR | 0.953 | 0.859 | 0.787 | 0.732 | 0.702 | 0.904 |
|  |  |  |  | - | 30 DSN + 400 VAR | 0.953 | 0.861 | 0.793 | 0.741 | 0.714 | 0.906 |
|  |  |  |  | - | 30 DSN + 500 VAR | 0.954 | 0.863 | 0.798 | 0.750 | 0.725 | 0.909 |
| HF | Beagle | Beagle | Beagle | - | 30 HF | 0.956 | 0.863 | 0.799 | 0.747 | 0.722 | 0.919 |
|  |  |  |  | - | 30 HF + 100 HF | 0.956 | 0.864 | 0.802 | 0.752 | 0.730 | 0.931 |
|  |  |  |  | - | 30 HF + 200 HF | 0.957 | 0.865 | 0.805 | 0.759 | 0.740 | 0.911 |
|  |  |  |  | - | 30 HF + 300 HF | 0.957 | 0.867 | 0.810 | 0.770 | 0.754 | 0.913 |
|  |  |  |  | - | 30 HF + 400 HF | 0.957 | 0.870 | 0.817 | 0.782 | 0.771 | 0.914 |
|  |  |  |  | - | 30 HF + 500 HF | 0.958 | 0.874 | 0.825 | 0.794 | 0.786 | 0.917 |
|  |  |  |  | - | 30 HF + 100 VAR | 0.955 | 0.860 | 0.791 | 0.731 | 0.703 | 0.920 |
|  |  |  |  | - | 30 HF + 200 VAR | 0.955 | 0.860 | 0.791 | 0.732 | 0.705 | 0.924 |
|  |  |  |  | - | 30 HF + 300 VAR | 0.956 | 0.861 | 0.795 | 0.741 | 0.716 | 0.907 |
|  |  |  |  | - | 30 HF + 400 VAR | 0.956 | 0.865 | 0.803 | 0.754 | 0.733 | 0.907 |
|  |  |  |  | - | 30 HF + 500 VAR | 0.956 | 0.868 | 0.809 | 0.764 | 0.747 | 0.909 |

**Supplementary Table 7:** Mean imputation accuracy with respect to the minor allele frequency of the sequenced DSN or HF animals. Imputation was performed for the different imputation setups including the comparison of 1) phasing strategies for the target population (unphased, phased with Beagle, phased with Eagle) and reference panel (phased with Beagle, phased with Eagle), 2) imputation software (Beagle and Minimac), 3) 1-step and 2-step imputation, and 4) adding successively HF animals or animals from various breeds (VAR) to the reference panel. The imputation accuracy was calculated using correlation (r) between observed and imputed genotypes for the minor allele frequency bins (0, 0.1], (0.1, 0.2] ,(0.2, 0.3] ,(0.3, 0.4] ,(0.4, 0.5].

| **1) Phasing of target population and reference panel** | | | | | | | | | | | |
| --- | --- | --- | --- | --- | --- | --- | --- | --- | --- | --- | --- |
| **Target population** | **Imputation software** | **Phasing of**  **target population** | **Phasing of**  **reference panel** | **Reference panel**  **(50k to 700k)** | **Reference panel**  **(to sequence level)** | **Mean r**  **MAF bin (0,0.1]** | **Mean r**  **MAF bin (0.1,0.2]** | **Mean r**  **MAF bin (0.2,0.3]** | **Mean r**  **MAF bin (0.3,0.4]** | **Mean r**  **MAF bin (0.4,0.5]** | **Mean r**  **overall** |
| DSN | Beagle | Beagle | Beagle | - | 30 DSN | 0.828 | 0.728 | 0.625 | 0.488 | 0.335 | 0.746 |
|  |  |  |  | - | 1000 bulls | 0.849 | 0.787 | 0.739 | 0.664 | 0.620 | 0.829 |
|  |  |  | Eagle | - | 30 DSN | 0.828 | 0.728 | 0.624 | 0.488 | 0.335 | 0.746 |
|  |  |  |  | - | 1000 bulls | 0.848 | 0.785 | 0.734 | 0.659 | 0.609 | 0.826 |
|  |  | Eagle | Beagle | - | 30 DSN | 0.828 | 0.727 | 0.624 | 0.487 | 0.333 | 0.745 |
|  |  |  |  | - | 1000 bulls | 0.848 | 0.787 | 0.736 | 0.663 | 0.616 | 0.828 |
|  |  |  | Eagle | - | 30 DSN | 0.828 | 0.728 | 0.623 | 0.487 | 0.333 | 0.745 |
|  |  |  |  | - | 1000 bulls | 0.848 | 0.785 | 0.733 | 0.660 | 0.607 | 0.826 |
|  |  | unphased | Beagle | - | 30 DSN | 0.828 | 0.727 | 0.624 | 0.487 | 0.334 | 0.745 |
|  |  |  |  | - | 1000 bulls | 0.852 | 0.795 | 0.750 | 0.680 | 0.635 | 0.836 |
|  |  |  | Eagle | - | 30 DSN | 0.828 | 0.728 | 0.623 | 0.487 | 0.333 | 0.745 |
|  |  |  |  | - | 1000 bulls | 0.851 | 0.793 | 0.746 | 0.675 | 0.625 | 0.834 |
|  | Minimac | Beagle | Beagle | - | 30 DSN | 0.822 | 0.736 | 0.633 | 0.524 | 0.439 | 0.757 |
|  |  |  |  | - | 1000 bulls | 0.843 | 0.771 | 0.707 | 0.619 | 0.549 | 0.805 |
|  |  |  | Eagle | - | 30 DSN | 0.822 | 0.735 | 0.627 | 0.512 | 0.423 | 0.753 |
|  |  |  |  | - | 1000 bulls | 0.837 | 0.758 | 0.685 | 0.584 | 0.500 | 0.788 |
|  |  | Eagle | Beagle | - | 30 DSN | 0.822 | 0.735 | 0.632 | 0.523 | 0.437 | 0.756 |
|  |  |  |  | - | 1000 bulls | 0.841 | 0.768 | 0.703 | 0.614 | 0.543 | 0.803 |
|  |  |  | Eagle | - | 30 DSN | 0.822 | 0.734 | 0.626 | 0.510 | 0.421 | 0.752 |
|  |  |  |  | - | 1000 bulls | 0.837 | 0.758 | 0.684 | 0.583 | 0.499 | 0.787 |
|  |  | unphased | Beagle | - | 30 DSN | 0.819 | 0.731 | 0.625 | 0.515 | 0.428 | 0.751 |
|  |  |  |  | - | 1000 bulls | 0.834 | 0.757 | 0.686 | 0.596 | 0.522 | 0.791 |
|  |  |  | Eagle | - | 30 DSN | 0.820 | 0.730 | 0.621 | 0.503 | 0.413 | 0.747 |
|  |  |  |  | - | 1000 bulls | 0.833 | 0.751 | 0.673 | 0.570 | 0.483 | 0.779 |
| HF | Beagle | Beagle | Beagle | - | 30 HF | 0.856 | 0.753 | 0.647 | 0.486 | 0.349 | 0.768 |
|  |  |  |  | - | 1000 bulls | 0.886 | 0.848 | 0.808 | 0.752 | 0.733 | 0.880 |
|  |  |  | Eagle | - | 30 HF | 0.856 | 0.754 | 0.649 | 0.490 | 0.355 | 0.768 |
|  |  |  |  | - | 1000 bulls | 0.884 | 0.844 | 0.803 | 0.744 | 0.722 | 0.875 |
|  |  | Eagle | Beagle | - | 30 HF | 0.856 | 0.752 | 0.646 | 0.484 | 0.346 | 0.767 |
|  |  |  |  | - | 1000 bulls | 0.884 | 0.844 | 0.804 | 0.743 | 0.726 | 0.877 |
|  |  |  | Eagle | - | 30 HF | 0.856 | 0.753 | 0.647 | 0.488 | 0.353 | 0.767 |
|  |  |  |  | - | 1000 bulls | 0.884 | 0.842 | 0.800 | 0.739 | 0.717 | 0.873 |
|  |  | unphased | Beagle | - | 30 HF | 0.856 | 0.752 | 0.646 | 0.484 | 0.346 | 0.767 |
|  |  |  |  | - | 1000 bulls | 0.897 | 0.868 | 0.838 | 0.787 | 0.777 | 0.898 |
|  |  |  | Eagle | - | 30 HF | 0.856 | 0.753 | 0.647 | 0.487 | 0.353 | 0.767 |
|  |  |  |  | - | 1000 bulls | 0.896 | 0.866 | 0.834 | 0.784 | 0.769 | 0.894 |
|  | Minimac | Beagle | Beagle | - | 30 HF | 0.838 | 0.733 | 0.627 | 0.497 | 0.415 | 0.758 |
|  |  |  |  | - | 1000 bulls | 0.872 | 0.811 | 0.747 | 0.660 | 0.606 | 0.838 |
|  |  |  | Eagle | - | 30 HF | 0.838 | 0.731 | 0.622 | 0.485 | 0.398 | 0.754 |
|  |  |  |  | - | 1000 bulls | 0.865 | 0.792 | 0.714 | 0.607 | 0.535 | 0.814 |
|  |  | Eagle | Beagle | - | 30 HF | 0.837 | 0.732 | 0.625 | 0.494 | 0.412 | 0.757 |
|  |  |  |  | - | 1000 bulls | 0.870 | 0.807 | 0.741 | 0.652 | 0.599 | 0.835 |
|  |  |  | Eagle | - | 30 HF | 0.838 | 0.729 | 0.620 | 0.483 | 0.395 | 0.752 |
|  |  |  |  | - | 1000 bulls | 0.865 | 0.790 | 0.711 | 0.604 | 0.532 | 0.812 |
|  |  | unphased | Beagle | - | 30 HF | 0.835 | 0.726 | 0.620 | 0.487 | 0.404 | 0.752 |
|  |  |  |  | - | 1000 bulls | 0.862 | 0.789 | 0.719 | 0.624 | 0.564 | 0.819 |
|  |  |  | Eagle | - | 30 HF | 0.836 | 0.725 | 0.616 | 0.476 | 0.388 | 0.748 |
|  |  |  |  | - | 1000 bulls | 0.861 | 0.779 | 0.696 | 0.585 | 0.510 | 0.802 |
| **2) Comparison of imputation software (Beagle versus Minimac)** | | | | | | | | | | | |
| **Target population** | **Imputation software** | **Phasing of**  **target population** | **Phasing of**  **reference panel** | **Reference panel**  **(50k to 700k)** | **Reference panel**  **(to sequence level)** | **Mean r**  **MAF bin (0,0.1]** | **Mean r**  **MAF bin (0.1,0.2]** | **Mean r**  **MAF bin (0.2,0.3]** | **Mean r**  **MAF bin (0.3,0.4]** | **Mean r**  **MAF bin (0.4,0.5]** | **Mean r**  **overall** |
| DSN | Beagle | Beagle | Beagle | - | 30 DSN | 0.826 | 0.725 | 0.624 | 0.477 | 0.319 | 0.737 |
|  |  |  |  | - | 1000 bulls | 0.843 | 0.774 | 0.716 | 0.634 | 0.583 | 0.811 |
|  | Minimac |  |  | - | 30 DSN | 0.818 | 0.712 | 0.612 | 0.490 | 0.404 | 0.734 |
|  |  |  |  | - | 1000 bulls | 0.838 | 0.756 | 0.685 | 0.582 | 0.514 | 0.786 |
| HF | Beagle | Beagle | Beagle | - | 30 HF | 0.838 | 0.734 | 0.631 | 0.485 | 0.349 | 0.752 |
|  |  |  |  | - | 1000 bulls | 0.867 | 0.820 | 0.780 | 0.724 | 0.696 | 0.858 |
|  | Minimac |  |  | - | 30 HF | 0.820 | 0.707 | 0.598 | 0.475 | 0.397 | 0.734 |
|  |  |  |  | - | 1000 bulls | 0.854 | 0.784 | 0.720 | 0.635 | 0.579 | 0.815 |
| **3) Comparison of 1-step and 2-step imputation** | | | | | | | | | | | |
| **Target population** | **Imputation software** | **Phasing of**  **target population** | **Phasing of**  **reference panel** | **Reference panel**  **(50k to 700k)** | **Reference panel**  **(to sequence level)** | **Mean r**  **MAF bin (0,0.1]** | **Mean r**  **MAF bin (0.1,0.2]** | **Mean r**  **MAF bin (0.2,0.3]** | **Mean r**  **MAF bin (0.3,0.4]** | **Mean r**  **MAF bin (0.4,0.5]** | **Mean r**  **overall** |
| DSN | Beagle | Beagle | Beagle | - | 30 DSN | 0.826 | 0.725 | 0.624 | 0.477 | 0.319 | 0.737 |
|  |  |  |  | - | 1000 bulls | 0.843 | 0.774 | 0.716 | 0.634 | 0.583 | 0.811 |
|  |  |  |  | 30 DSN | 30 DSN | 0.810 | 0.691 | 0.570 | 0.397 | 0.251 | 0.709 |
|  |  |  |  | 30 DSN | 1000 bulls | 0.769 | 0.661 | 0.568 | 0.438 | 0.310 | 0.712 |
|  |  |  |  | 1000 bulls | 30 DSN | 0.813 | 0.716 | 0.630 | 0.515 | 0.438 | 0.759 |
|  |  |  |  | 1000 bulls | 1000 bulls | 0.782 | 0.728 | 0.670 | 0.589 | 0.533 | 0.778 |
| HF | Beagle | Beagle | Beagle | - | 30 HF | 0.838 | 0.734 | 0.631 | 0.485 | 0.349 | 0.752 |
|  |  |  |  | - | 1000 bulls | 0.867 | 0.820 | 0.780 | 0.724 | 0.696 | 0.858 |
|  |  |  |  | 30 HF | 30 HF | 0.824 | 0.702 | 0.573 | 0.407 | 0.291 | 0.725 |
|  |  |  |  | 30 HF | 1000 bulls | 0.795 | 0.678 | 0.581 | 0.452 | 0.353 | 0.734 |
|  |  |  |  | 1000 bulls | 30 HF | 0.834 | 0.748 | 0.673 | 0.576 | 0.527 | 0.794 |
|  |  |  |  | 1000 bulls | 1000 bulls | 0.827 | 0.782 | 0.744 | 0.679 | 0.649 | 0.831 |
|  |  |  |  |  |  |  |  |  |  |  |  |
|  |  |  |  |  |  |  |  |  |  |  |  |
|  |  |  |  |  |  |  |  |  |  |  |  |
| **4) Adding HF animals or animals from various breeds to the reference panel** | | | | | | | | | | | |
| **Target population** | **Imputation software** | **Phasing of**  **target population** | **Phasing of**  **reference panel** | **Reference panel**  **(50k to 700k)** | **Reference panel**  **(to sequence level)** | **Mean r**  **MAF bin (0,0.1]** | **Mean r**  **MAF bin (0.1,0.2]** | **Mean r**  **MAF bin (0.2,0.3]** | **Mean r**  **MAF bin (0.3,0.4]** | **Mean r**  **MAF bin (0.4,0.5]** | **Mean r**  **overall** |
| DSN | Beagle | Beagle | Beagle | - | 30 DSN | 0.826 | 0.725 | 0.624 | 0.477 | 0.319 | 0.737 |
|  |  |  |  | - | 30 DSN + 100 HF | 0.811 | 0.694 | 0.567 | 0.404 | 0.296 | 0.706 |
|  |  |  |  | - | 30 DSN + 200 HF | 0.806 | 0.683 | 0.554 | 0.402 | 0.301 | 0.702 |
|  |  |  |  | - | 30 DSN + 300 HF | 0.807 | 0.686 | 0.565 | 0.417 | 0.323 | 0.709 |
|  |  |  |  | - | 30 DSN + 400 HF | 0.808 | 0.690 | 0.576 | 0.432 | 0.346 | 0.717 |
|  |  |  |  | - | 30 DSN + 500 HF | 0.810 | 0.696 | 0.588 | 0.452 | 0.367 | 0.726 |
|  |  |  |  | - | 30 DSN + 500 VAR | 0.821 | 0.714 | 0.598 | 0.434 | 0.307 | 0.715 |
|  |  |  |  | - | 30 DSN + 100 VAR | 0.819 | 0.711 | 0.595 | 0.434 | 0.315 | 0.715 |
|  |  |  |  | - | 30 DSN + 200 VAR | 0.820 | 0.712 | 0.600 | 0.449 | 0.336 | 0.721 |
|  |  |  |  | - | 30 DSN + 300 VAR | 0.821 | 0.716 | 0.609 | 0.467 | 0.360 | 0.729 |
|  |  |  |  | - | 30 DSN + 400 VAR | 0.823 | 0.721 | 0.619 | 0.483 | 0.384 | 0.737 |
| HF | Beagle | Beagle | Beagle |  | 30 HF | 0.838 | 0.734 | 0.631 | 0.485 | 0.349 | 0.752 |
|  |  |  |  | - | 30 HF + 100 HF | 0.839 | 0.737 | 0.638 | 0.500 | 0.385 | 0.757 |
|  |  |  |  | - | 30 HF + 200 HF | 0.840 | 0.739 | 0.643 | 0.513 | 0.407 | 0.762 |
|  |  |  |  | - | 30 HF + 300 HF | 0.841 | 0.743 | 0.653 | 0.534 | 0.438 | 0.771 |
|  |  |  |  | - | 30 HF + 400 HF | 0.842 | 0.749 | 0.665 | 0.558 | 0.474 | 0.781 |
|  |  |  |  | - | 30 HF + 500 HF | 0.844 | 0.755 | 0.678 | 0.580 | 0.508 | 0.791 |
|  |  |  |  | - | 30 HF + 100 VAR | 0.834 | 0.726 | 0.610 | 0.448 | 0.332 | 0.732 |
|  |  |  |  | - | 30 HF + 200 VAR | 0.833 | 0.724 | 0.607 | 0.449 | 0.340 | 0.732 |
|  |  |  |  | - | 30 HF + 300 VAR | 0.834 | 0.727 | 0.612 | 0.465 | 0.361 | 0.738 |
|  |  |  |  | - | 30 HF + 400 VAR | 0.836 | 0.734 | 0.628 | 0.491 | 0.395 | 0.750 |
|  |  |  |  | - | 30 HF + 500 VAR | 0.837 | 0.739 | 0.640 | 0.511 | 0.423 | 0.759 |
